# Beneficial effect of low-dose radioiodine ablation for Graves’ orbitopathy: results of a retrospective study

**DOI:** 10.1007/s40618-021-01544-1

**Published:** 2021-04-12

**Authors:** Giulia Lanzolla, Francesca Menconi, Francesca Nicolì, Chiara Posarelli, Maria Novella Maglionico, Michele Figus, Marco Nardi, Claudio Marcocci, Michele Marinò

**Affiliations:** 1 Department of Clinical and Experimental Medicine, Endocrinology Unit II, University of Pisa and University Hospital of Pisa, Via Paradisa 2, 56124 Pisa, Italy; 2 Department of Surgical, Medical and Molecular Pathology, Ophthalmopathy Unit I, University of Pisa and University Hospital of Pisa, Via Paradisa 2, 56124 Pisa, Italy

**Keywords:** Graves’ disease, Graves’ orbitopathy, Ablation, Radioiodine, Thyroid, Autoimmunity

## Abstract

**Objective:**

Graves’ orbitopathy (GO) reflects an autoimmune response against antigens expressed by the thyroid and orbital tissues. Elimination of thyroid antigens may be beneficial for GO. Total thyroid ablation (TTA) [thyroidectomy (Tx), followed by 30 mCi of radioiodine] was shown to exert a beneficial effect on GO following intravenous glucocorticoids (ivGC) compared with Tx alone. Here, we investigated retrospectively whether TTA performed with a 15 mCi of radioiodine still maintains advantages over Tx.

**Methods:**

Thirty-two subjects, 13 treated with TTA (performed with 15 mCi of radioiodine) and 19 with Tx alone, all with moderately severe, active GO, treated with ivGC, were studied. The primary objective was the outcome of GO at 24 weeks based on a composite evaluation.

**Results:**

The two groups did not differ at baseline in terms of sex, age, smoking habits, TSH, anti-TSH receptor autoantibodies, GO duration and eye features. The proportion of GO responders at 24 weeks was greater in the TTA (61.5%) than in the Tx group (26.3%, *P* = 0.046). In contrast, GO outcome at 48 weeks did not differ between the two groups (69.2% vs 52.6% of responder in TTA and Tx group, respectively). The outcome of the individual GO features did not differ between the two groups both a 24 and 48 months.

**Conclusions:**

The advantage of total thyroid ablation seems to be a more rapid response for GO to ivGC treatment. Prospective, randomized studies in a larger number of subjects are needed to confirm our findings.

## Introduction

Graves’ orbitopathy (GO) is believed to be due to an autoimmune reaction against antigens expressed by thyroid epithelial cells and orbital tissues, being the TSH receptor (TSH-R) the most suitable candidate, and the insulin-like growth factor-1 receptor a putative one [1–3]. Based on this hypothesis, it has been postulated that elimination of thyroid antigens may be beneficial for GO [4]. A few studies have indeed shown a beneficial effect of total thyroid ablation (TTA), achieved by near-total thyroidectomy (Tx), followed by radioiodine given as a fixed activity of 30 mCi [5, 6]. In a recent, pilot, prospective study, we showed that a sufficient extent of thyroid ablation, assessed as recombinant human TSH (rhTSH)-stimulated Tg, can be achieved with a radioiodine activity of 15 mCi upon rhTSH stimulation [7]. Therefore, over the last few years, we treated a few thyroidectomized patients with moderately severe and active GO with 15 mCi of radioiodine, followed by intravenous glucocorticoids (ivGC), the usual treatment for this obnoxious eye disease [8]. In two previous, randomized clinical trials in which GO patients who underwent ivGC following TTA (performed with a 30 mCi activity after surgery) or Tx alone were compared, TTA resulted in a beneficial effect on GO [5, 6]. Thus, the aim of the present study was to investigate retrospectively whether TTA performed with a 15 mCi activity of radioiodine still maintains advantages over Tx alone following ivGC. The results show a better outcome of GO at 24 weeks, assessed using a composite evaluation, in patients treated with TTA, in confirmation of the validity of this therapeutic approach.

## Subjects and methods

### Study design

The study was aimed at investigating retrospectively the outcome of moderately severe, active GO following ivGC, based on thyroid treatment, namely TTA vs Tx alone. The study entailed the inclusion of all consecutive patients with moderately severe, active GO who had undergone ivGC treatment following either Tx or TTA, the latter performed with 15 mCi of radioiodine after thyroidectomy, over a period of 2 consecutive years.

### Setting

The study was performed in a tertiary referral center, namely the University Hospital of Pisa. A database search was conducted to identify patients with moderately severe GO treated with ivGC following either Tx or TTA from January 1st 2018 till December 31st 2019. Patients were included by means of consecutive sampling. The inclusion and exclusion criteria are reported below. The data were recorded in a database. The following database validation procedures were employed: allowed character checks, batch totals, missing records check, cardinality check, digits check, consistency check, control totals, cross-system consistency check, data type check, hash totals, limit check, logic check, presence check, range check, spelling and grammar check, and uniqueness check.

### Participants

Inclusion criteria were: (1) male and female patients aged 18–85 years; (2) a diagnosis of Graves’ hyperthyroidism (GH) before thyroidectomy, based on a history of hyperthyroidism, associated with previous or present detectable serum autoantibodies against the TSH-R (TRAbs); (3) near-total thyroidectomy; (4) a diagnosis of moderately severe, active GO, based on the presence of at least one of the following eye features, associated with a clinical activity score (CAS) ≥ 3/7: exophthalmometry ≥ 22 mm; eyelid aperture ≥ 13 mm; (iii) presence of inconstant or constant diplopia; (5) written, signed informed consent to data use.

Exclusion criteria were: (1) treatment with GC or any immunosuppressive medication in the preceding 3 months; (2) presence of optic neuropathy, according to previously established criteria [8]; (3) lack of informed consent.

A total of 32 subjects (10 men and 22 women, age 47.5 ± 10.5 years, range 19–64 years) who satisfied the inclusion criteria and evaded the exclusion criteria were recruited, of whom 13 had been treated with TTA and 19 with Tx alone. They were enrolled in the study and evaluated at baseline and then at 24 and 48 weeks. Being a retrospective study, no ethical approval was required.

### Outcomes

The primary objective of the study was the overall outcome of GO at 24 weeks based on a composite evaluation. Patients were considered as responders when at least two of the following criteria were fulfilled, without worsening of the other criteria: (1) reduction in proptosis ≥ 2 mm in at least one eye, with no increase ≥ 2 mm in the other eye; (2) reduction of CAS ≥ 2/7 points; (3) reduction in eyelid aperture ≥ 2 mm in at least one eye, with no increase ≥ 2 mm in the other eye; (4) disappearance or improvement (change of degree from constant to inconstant or intermittent, or from inconstant to intermittent) of diplopia. In all other cases, patients were considered as non-responders.

The secondary outcomes were: (1) the overall outcome of GO at 48 weeks; (2) the outcome of proptosis at 24 and 48 weeks, namely a reduction ≥ 2 mm in at least one eye, with no increase ≥ 2 mm in the other eye; 2) the outcome of CAS at 24 and 48 weeks, namely a reduction ≥ 2/7 points; (3) the outcome of eyelid aperture at 24 and 48 weeks, namely a reduction ≥ 2 mm in at least one eye, with no increase ≥ 2 mm in the other eye; (4) the outcome of diplopia at 24 and 48 weeks, namely its disappearance or change in degree from constant to inconstant or intermittent, or from inconstant to intermittent.

### Sources of data and measurements

An ophthalmological evaluation was performed in all patients, including: (1) exophthalmometry; (2) measurement of eyelid aperture; (3) evaluation of CAS; (4) assessment of diplopia; (5) assessment of the corneal status; (6) examination of the fundi; and (7) measurement of visual acuity. The following blood tests were performed in all subjects: (1) FT4, FT3 (Vitros Immunodiagnostics, Raritan, NJ); (2) TSH (Immulite 2000, Siemens Healthcare, Gwynedd, UK); and (3) anti-TSH receptor autoantibodies (TRAbs) (Brahms, Berlin, Germany).

### Interventions

All patients had been subjected to near-total thyroidectomy. Ablation was performed in 13 patients with 15 mCi of ^131^I upon rhTSH stimulation. A single dose of 0.9 mg was administered intramuscularly 48 and 24 h before radioiodine. All patients were on LT4 therapy at replacement doses. All patients were treated with ivGC after thyroid treatment. Patients were given 1 iv infusion/week of methylprednisolone for a total of 12 infusions. The dose was 500 mg for the first six infusions and 250 mg for the last six infusions, for a cumulative dose of 4.5 g, as described previously [7]. No major side effects of GC were observed.

### Statistical power

As reported above, we identified 32 patients who fulfilled the inclusion criteria and evaded the exclusion criteria during the indicated period. Being a retrospective study over a limited period of time, a sample size could not be calculated. Instead, based on the results of the primary outcome measure (see below), we calculated the post-hoc statistical power, which resulted to be 47.6% for a* P* value ≤ 0.05.

### Quantitative variables

Continuous variables with a normal distribution, which was assessed using the Shapiro–Wilks test, are presented as mean ± SD. The remaining continuous variables are presented as median and IQR.

### Statistical analyses

When appropriate, the following tests were performed: (1) ANOVA with Bonferroni’s correction; (2) Mann–Whitney; and (3) Chi-square.

## Results

### Features of patients

The main demographic and clinical variables of the patient population at baseline are reported in Table 1. The two groups did not differ in terms of gender, age, smoking habits, TSH and TRAbs concentrations at baseline, GO duration and time between Tx and baseline evaluation. Likewise, the eye features of the two groups were similar, having both a moderately severe, active GO.

**Table 1:** Features of patients with Graves’ Orbitopathy (GO) at baseline

Feature	TTA	TX	*P*
Gender	Males: 2; Females: 11	Males: 8; Females: 11	0.109
Age (yr)	48 ± 9.7 (range 26–60)	47.2 ± .11.3 (range 19–64)	NS
Smoking habits	Non-smokers 6 Ex smokers: 2 Current smokers: 5	Non-smokers 7 Ex smokers: 3 Current smokers: 9	0.888
TSH (mU/L; NV 0.4–4)	1.1 (IQR 0.3–3.5)	2.0 (IQR 0.2–4.5)	NS
TRAbs (IU/L; NV < 1.5)	9.3 (IQR 2.6–14.2)	4.4 (IQR 2–8.5)	NS
GO duration (mo.)	15 (IQR 15–18)	13 (IQR 9–16.5)	NS
Time between TX and baseline evaluation (mo.)	4 (IQR 4–6)	4 (IQR 2.5–7.5)	NS
Exophthalmometry (most affected eye) (mm)	24.7 ± 2.1 (range 19–28)	24.9 ± 3.6 (range 19–35)	NS
Clinical Activity Score (points)	4.6 ± 1.3 (range 3–7)	4.2 ± 0.9 (range 3–6)	NS
Double vision	Absent: 2 Intermittent: 1 Inconstant: 4 Constant: 6	Absent: 6 Intermittent: 1 Inconstant: 9 Constant: 3	NS
Best corrected visual acuity (most affected eye) (decimals; NV 1.0)	0.9 ± 0.02 (range 0.9–1.0)	1 ± 0 (range 10–10)	NS

*TTA* total thyroid ablation (thyroidectomy followed by 15 mCi of radioiodine),* Tx* thyroidectomy, *TRAbs* serum anti-TSH-receptor autoantibodies, *NV* normal values

Continuous variables are reported as mean ± SD or median and IQR

*P* values were obtained by Chi square (categorical variables), ANOVA with Bonferroni’s correction (continuous variables with normal distribution), or Mann–Whitney (continuous variables with non-normal distribution)

### Outcome measures

**Fig. 1:**
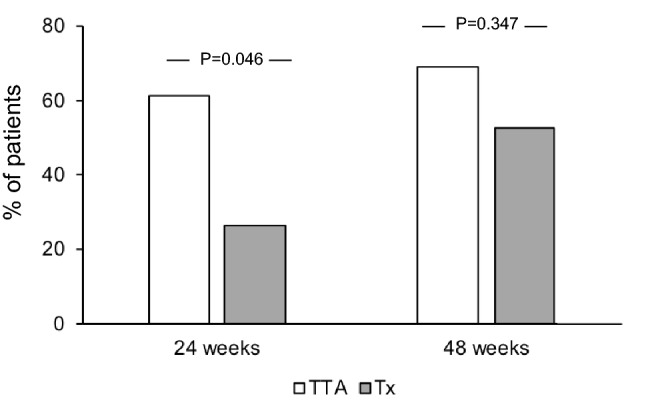
Overall outcome of Graves’ Orbitopathy (GO) (proportion of responders) at 24 and 48 weeks, following intravenous glucocorticoids, in patients with moderately severe, active GO treated with total thyroid ablation (TTA, thyroidectomy followed by 15 mCi of radioiodine), or thyroidectomy alone (Tx). Patients were considered as responders when at least two of the following criteria were fulfilled, without worsening of the other criteria: (1) reduction in proptosis ≥ 2 mm in at least one eye, with no increase ≥ 2 mm in the other eye; (2) reduction of Clinical Activity Score ≥ 2/7 points; (3) reduction in eyelid aperture ≥ 2 mm in at least one eye, with no increase ≥ 2 mm in the other eye; (4) disappearance or improvement (change of degree from constant to inconstant or intermittent, or from inconstant to intermittent) of diplopia. P values were obtained by Chi-square

**Fig. 2:**
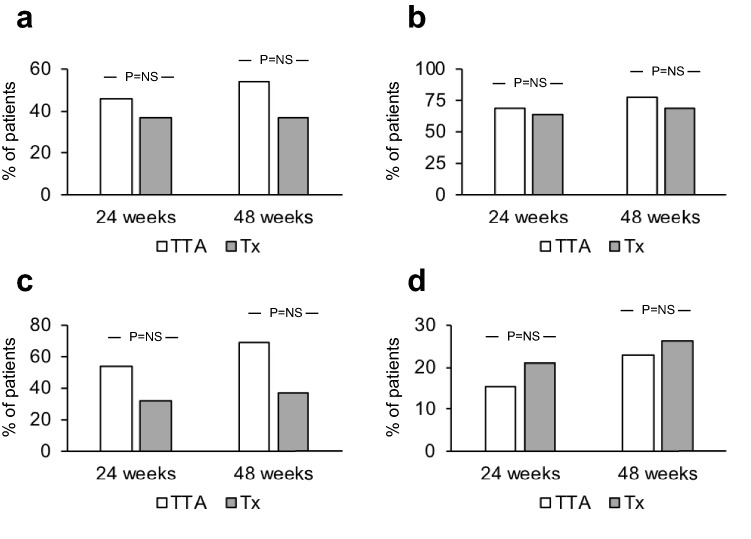
Outcome of individual eye features of Graves’ Orbitopathy (GO) (proportion of responders) at 24 and 48 weeks, following intravenous glucocorticoids, in patients with moderately severe, active GO treated with total thyroid ablation (TTA, thyroidectomy followed by 15 mCi of radioiodine), or thyroidectomy alone (Tx).** a** Proptosis. Patients with a reduction ≥ 2 mm in at least one eye and with no increase ≥ 2 mm in the other eye were considered as responder; **b** Clinical Activity Score. Patients with a reduction ≥ 2/7 points were considered as responder; **c** Diplopia. Patients with disappearance or improvement (change of degree from constant to inconstant or intermittent, or from inconstant to intermittent) were considered as responders; **d** Eyelid aperture. Patients with a reduction ≥ 2 mm in at least one eye and with no increase ≥ 2 mm in the other eye were considered as responders. P values were obtained by Chi-square

As shown in Fig. 1, using a composite evaluation, the proportion of GO responders to ivGC at 24 weeks was greater in the TTA (61.5%) than in the Tx group (26.3%), with a statistically significant difference (*P* = 0.046), thereby fulfilling the primary outcome of the study. In contrast, GO outcome at 48 weeks did not differ significantly between the two groups, in spite of a slightly greater proportion of responders in the TTA group (69.2% vs 52.6% in the Tx group). As shown in Fig. 2, the outcome of the individual GO features did not differ between patients who underwent TTA and those who underwent Tx, in spite of a tendency towards a better response to treatment in the TTA group in terms of diplopia (Fig. 2c), which did not reach statistical significance, possibly because of the relatively limited number of subjects and the consequent relatively low statistical power.

## Discussion

The optimal treatment of thyroid dysfunction in patients with GO as well as its impact on the eye disease remain to be established [8]. Whereas some prefer a conservative approach based on the prolonged use of anti-thyroid medications, other have proposed an ablative approach with Tx, radioiodine or both, based on the idea that GO reflects an autoimmune response against antigens expressed both in the thyroid and in fibroadipose orbital tissues [1, 4, 8]. TTA, performed with Tx followed by radioiodine, was first proposed by Catz and Perzik [9], and two retrospective studies showed an improvement of GO following this therapeutic approach [10, 11]. Two randomized clinical trials conducted in patients with moderately severe, active GO treated with ivGC following Tx alone or TTA, the latter performed with a 30 mCi activity of radioiodine [5, 6], showed a better outcome of GO in patients who had undergone TTA. In a relatively recent study, we demonstrated that a 15 mCi activity of radioiodine upon rhTSH stimulation is equally effective as a 30 mCi activity in achieving thyroid ablation in patients with Graves’ hyperthyroidism following thyroidectomy, as shown by rhTSH-stimulated Tg assays [7]. Thus, here, we investigated retrospectively whether TTA performed with a 15 mCi activity of radioiodine is followed by a better outcome of GO to ivGC compared with thyroidectomy alone. Our results show that ablation performed as here is associated with a better overall outcome of GO to ivGC in the short term (24 weeks), whereas in the long term (48 weeks), ablation did not offer a substantial advantage compared with thyroidectomy alone. Our findings are in line with the previous observations in GO patients given a 30 mCi activity of radioiodine, who displayed a better GO outcome in the short [5], but not in the long term [12]. Thus, the real advantage of total thyroid ablation seems to be a more rapid response to ivGC treatment. The better outcome of GO at 24 weeks in TTA patients probably reflected a greater extent of amelioration of proptosis and diplopia, even though neither of the variables were significantly different compared with the Tx group, presumably because of the relatively small numerosity of our sample size, with a consequent, relatively low statistical power. The latter, was certainly one of the major limitations of the present study, along with its retrospective nature. Thus, prospective, possibly randomized studies in a larger number of subjects are certainly needed to confirm these preliminary findings.
